# Supplementary material for: Rapid and Visual Detection of SARS-CoV-2 RNA Based on Reverse Transcription-Recombinase Polymerase Amplification with Closed Vertical Flow Visualization Strip Assay
Source: Microbiol Spectr. 2023 Jan 9;11(1):e02966-22. doi: 10.1128/spectrum.02966-22 (PMC9927448; doi:10.1128/spectrum.02966-22)
Supplement: Supplemental file 1 — Fig. S1. Download spectrum.02966-22-s0001.pdf, PDF file, 0.9 MB [file spectrum.02966-22-s0001.pdf]

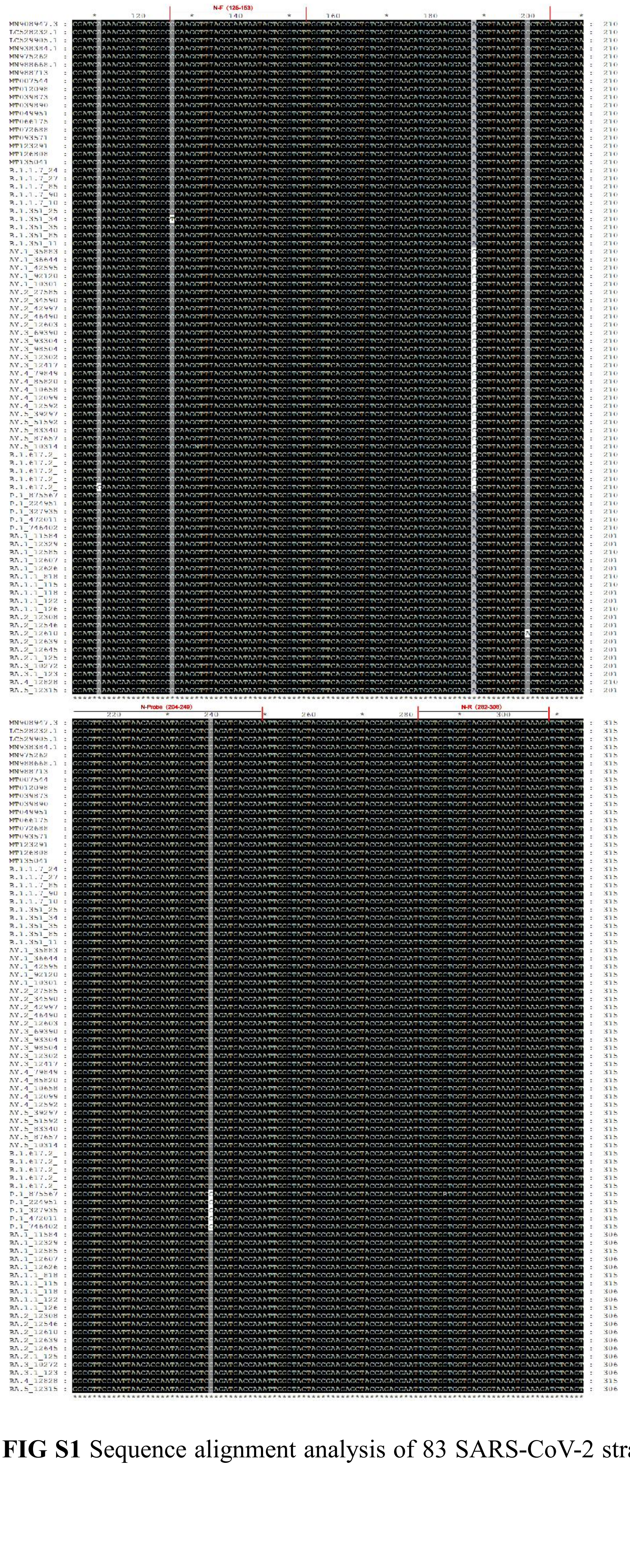

FIG S1 Sequence alignment analysis of 83 SARS-CoV-2 strains. All SARS-CoV-2 *N*

genes were retrieved from GenBank and GISIS, and the sequence alignment was analyzed by Genedoc. The primers and probe used in this study were labeled.
